# Medical cannabis dimethyl ether, ethanol and butane extracts inhibit the *in vitro* growth of bacteria and dermatophytes causing common skin diseases

**DOI:** 10.3389/fmicb.2022.953092

**Published:** 2022-09-20

**Authors:** Tomáš Skala, Zdeˇnka Kahánková, Jan Tauchen, Anežka Janatová, Pavel Klouˇcek, Vít Hubka, Adéla Fraˇnková

**Affiliations:** ^1^Department of Food Science, Czech University of Life Sciences Prague, Prague, Czechia; ^2^Department of Botany, Faculty of Science, Charles University, Prague, Czechia

**Keywords:** extraction method, *Cannabis sativa*, antimicrobial activity, dermatophytes, dimethyl ether, skin infection

## Abstract

Cannabis preparations are gaining popularity among patients with various skin diseases. Due to the lack of scientific evidence, dermatologists remain cautious about their prescriptions. So far, only a few studies have been published about the effects of high-potency cannabis extracts on microorganisms (especially dermatophytes) causing skin problems that affect more than 25% of the worldwide population. Even though, the high-potency cannabis extracts prepared by cold extraction are mostly composed of non-psychoactive tetrahydrocannabinolic acid (THCA) and only low amount of THC, their use in topical treatment can be stigmatized. The *in vitro* antimicrobial and antifungal activity of two high potent cannabis strains extracted by three solvents traditionally or currently used by cannabis users (ethanol; EtOH, butane; BUT, dimethyl ether; DME) was investigated by broth dilution method. The chemical profile of cannabis was determined by high-performance liquid chromatography with ultraviolet detection and gas chromatography with mass spectrometer and flame ionization detector. The extraction methods significantly influenced chemical profile of extracts. The yield of EtOH extracts contained less cannabinoids and terpenes compared to BUT and DME ones. Most of the extracts was predominantly (>60%) composed of various cannabinoids, especially THCA. All of them demonstrated activity against 18 of the 19 microorganisms tested. The minimal inhibitory concentrations (MICs) of the extracts ranged from 4 to 256 μg/mL. In general, the bacteria were more susceptible to the extracts than dermatophytes. Due to the lower content of biologically active substances, the EtOH extracts were less effective against microorganisms. Cannabis extracts may be of value to treat dermatophytosis and other skin diseases caused by various microorganisms. Therefore, they could serve as an alternative or supportive treatment to commonly used antibiotics.

## Introduction

Human is a unique organ inhabited by a diverse collection of bacteria, fungi, and viruses where the microorganisms usually live in homeostasis (Schommer and Gallo, 2013). Dysbiosis of the microbiome can lead to the development of various skin diseases, which are understood as the 4th most common contributors to the global burden of non-fatal diseases worldwide (Hay et al., 2014). Among them, skin infections of fungal and bacterial origin are the most common ones. Bacterial infections are commonly caused by, e.g., S*taphylococcus epidermidis*, *S. aureus*, and *Streptococcus pyogenes.* These opportunistic pathogens can cause serious infections, such as endocarditis, sepsis, or streptococcal toxic shock syndrome. However, more frequently they cause painful skin infections such as folliculitis, impetigo, etc. (Johansson et al., 2010; Becker et al., 2014).

The superficial fungal diseases (dermatomycosis) are predominantly caused by *Epidermophyton*, *Microsporum* or *Trichophyton spp*. It is estimated that 20–25% of the global population suffer with them (Havlickova et al., 2008; Panda and Verma, 2017) and due to globalization, the number of the cases of dermatophytosis is increasing (Begum et al., 2020). For example, the prevalence of dermatophytosis in India is greater than 50% (Ramaraj et al., 2016). Although the symptoms of fungal infection are generally mild and painless, their psychological and social burden on patients can be significant (Rajagopalan et al., 2018).

Nowadays, bacterial and fungal skin diseases are treated with topical or systemic antibiotics. Unfortunately, increasing antibiotic and antifungal resistance is becoming a significant worldwide problem. For example, 17–32% of clinical *Trichophyton* isolates in India were identified to be resistant to the widely used antibiotic terbinafine (TB) (Singh et al., 2018). The situation is even more alarming in the case of bacteria, as the increasing number of multidrug-resistant strains such as Methicillin-resistant *S. aureus* (MRSA) (Alves et al., 2018) is identified every year. The international strategies to tackle the antimicrobial resistance are known and slowly adopted by countries worldwide (WHO, 2019). However, there is no doubt that the discovery of new agents with antibiotic activity remains of great importance.

*Cannabis sativa*, after its legalization for medicinal purposes, is in the scope of many researches. It has been used in traditional medicine for various purposes including treatment of skin infections (Zuardi, 2006). Currently, cannabis is recommended for the treatment of various dermatologic conditions, i.e., psoriasis, lupus or acne, however, the scientific evidence on its activity is limited (Dhadwal and Kirchhof, 2018; Lim and Kirchhof, 2019).

Its positive effects on various diseases are attributed to cannabinoids and terpenes. Among other biological activities, antimicrobial action has been described for all of them, especially for cannabigerol (CBG), detla-9-tetrahydrocannabinol (THC), cannabigerolic acid (CBGA), and detla-9-tetrahydrocannabiolic acid (THCA) (Farha et al., 2020; Pasquali et al., 2020; Blaskovich et al., 2021) and also for most of the abundant terpenes – myrcene, limonene, β-pinene and linalool (Pepeljnjak et al., 2005; Sanguinetti et al., 2007; Singh et al., 2010). Moreover, it has been demonstrated that the synergistic effect between cannabinoids and terpenes may contribute to more effective treatment (Russo, 2011).

Unfortunately, high-potency cannabis is mainly associated with its psychoactive potential caused by THC, which is formed from THCA during the thermal processing of the naïve extract. At the same time, THCA is non-psychoactive cannabinoid with anti-inflammatory, anticancer and neuroprotective effect. For the topical application cannabis is currently prescribed in the form of dried flowers, creams, tinctures, or extracts (Zajicek et al., 2012; Peschel, 2016; Palmieri et al., 2019). Even though the extracts added to the preparations are usually not thermally processed, its topical application can be stigmatized and not acceptable for many people. Moreover, increasing trend in the use of cannabis extracts that are not heated can be observed among people (Anderson et al., 2019). The efficiency of each preparation is influenced not only by the cannabis variety (chemotype) but also by the type of extraction method, solvent used, and final formulation. Organic solvents with different polarities such as methanol, ethanol, acetone, or hexane are the most commonly used for the preparation of cannabis extracts (Wang et al., 2018). The solvent extraction is very simple; on the other hand, the solvent evaporation can be demanding.

For this reason, cannabis concentrates prepared by gases are gaining popularity among recreational users and medicinal marijuana patients (Daniulaityte et al., 2017). Supercritical CO_2_ extraction of cannabis become widely accepted technique in industry (Gallo-Molina et al., 2019). While recreational users prefer to use butane (Chan et al., 2017) and quite recently dimethyl ether (DME) for the extraction. Both gases are highly inflammable, butane is also highly toxic and can contain various impurities (Al-Zouabi et al., 2018). On the other hand, DME is understood to be of low toxicity and is also used in food industry for extraction of animal protein (EFSA, 2009). Therefore. could become one of the alternative techniques for cannabis extraction. So far, only a limited number of studies have investigated the influence of extraction techniques on the activity of cannabis extract. Moreover, although cannabis contains two groups of biologically active substances with high antimicrobial potential, its role in the treatment of bacterial or superficial fungal infections has also not been intensively investigated so far. Therefore, the aim of our research was to evaluate the antimicrobial and antifungal activity of two medicinal cannabis strains extracted by various methods against selected bacteria and dermatophytes.

## Materials and methods

### Chemicals

All solvents used for the HPLC and GC analysis were of analytical grade. Acetonitrile (ACN) and formic acid (FA) together with terpene standards [(+)-3-carene, camphene, α-pinene, ß-myrcene, (+)-limonene, terpinolene, linalool, fenchol, α-terpineol, caryophyllene, α-bergamotene, humulene, farnesene mix, caryophyllene oxide] and n-alkane standard were purchased from Sigma-Aldrich (Prague, Czechia). Methanol, n-hexane, dimethyl sulfoxide (DMSO), and ethanol (EtOH) were purchased from VWR Chemicals (Prague, Czechia). Standards of cannabinoids, namely, cannabidivarin (CBDV), cannabidivarinic acid (CBDVA), cannabigerol (CBG), cannabigerolic acid (CBGA), cannabinol (CBN), cannabinolic acid (CBNA), cannabidiol (CBD), cannabidiolic acid (CBDA), cannabichromene (CBC), tetrahydrocannabivarin (THCV), Δ^9^-tetrahydrocannabinol (THC), and tetrahydrocannabinolic acid A (THCA-A) were obtained from Cayman Chemicals (Ann Arbor, United States). Extraction gas butane (BUT), and dimethylether (DME) were provided by RSonic (Berlin, DE) and Dexso GmbH (Pratteln, CH), respectively. Microbiological growth media Mueller-Hinton Broth (MHB), Sabourad dextrose agar (SDA) were bought from OXOID (Prague, Czechia), horse defibrinated blood was purchased from Thermo Fisher Scientific (Waltham, United States), antibiotics clotrimazole (CLT), chloramphenicol (CLP), ampicilin (AMP), terbinafine (TB), and RPMI 1640 medium from Sigma-Aldrich.

### Plant material and extract preparation

The dried inflorescences from two cannabis genotypes – Forbidden Fruit (FF) (Czech Seed Bank, Czechia) and Chocolope (CHP) (DNA Genetics, NL) were used for the preparation of extracts. The plants were cultivated under controlled indoor conditions at the Department of Food Science, Faculty of Agrobiology Food and Natural Resources, Czech University of Life Sciences in Prague in 2020. Each genotype was cultivated on the table (2 m^2^) equipped with drip irrigation system. Plants were placed in pots containing Euro Pebbles (expanded clay) as growth medium. Air ventilation unit maintained the room temperature and humidity between 22 and 30°C, and 40 and 70%, respectively. The microclimatic conditions were adjusted according to the plant growth phase. Double-ended high-pressure sodium lamps were used to provide a suitable spectrum of light at a power of 1,000 W. The detailed information about plant nutrition were described previously by Janatová et al. (2018). Each cannabis genotype was extracted by three types of solvents – ethanol (EtOH), butane (BUT), and dimethyl ether (DME). To prepare first type of extract, 60 g of dried homogenized cannabis inflorescences were macerated for 48 h in 96% ethanol in the ratio 6:1 (solvent: flower; v/w). Subsequently, the extract was filtered, and the solvent evaporated using a Rotavapor^®^ R-100 vacuum evaporator (Buchi, CHE) at 40°C. The Dexso extractor was used for the preparation of butane and dimethyl ether extracts. The extractor was filled with 30 g of plant material to which 500 mL bottle containing compressed BUT or DME was connected. The gas flow was allowed to enter, and the extract was collected at the bottom to the teflon paper. To remove the residues of the gases, the extracts were placed for 2 h under the vacuum at Glass Vac (BVV, United States).

### Determination of the cannabinoid content in extracts by high-performance liquid chromatography with ultraviolet detection

Ten milligrams of each extract were dissolved in 1 mL of MeOH, filtered through 0.45 μm nylon filters (Agilent, United States), transferred to a HPLC vial, and submitted for analysis.

The apparatus consisted of UltiMate 3000 HPLC system (Thermo Fisher Scientific, United States) and was equipped with UV detector. Cannabinoids analysis was performed on an Excel SuperC18 column (250 × 4.6 mm, 3 μm, 90 A; ACE, Scotland). Compounds were separated using a gradient elution employing water + 0.075% FA (A) and ACN + 0.5% FA (B) as mobile phase under following gradient (A:B): 0′, 30:70; 1′, 30:70; 10′, 0:100; 12′, 0:100; 14′, 30:70. Temperature of the column compartment was set at 40°C. Injection was 10 μL and flow rate was 1 mL/min. Cannabinoids were detected at wavelengths between 190 and 400 nm. Quantification was done under 210 nm. The evaluation of the acquired data was performed using the Chromeleon 7.2 software (Thermo Fisher Scientific, United States). Standard calibration curves were prepared in a concentration range of 2–100 μg/mL with six concentration levels (100, 50, 20, 10, 5, and 2 μg/mL). UV peak areas of the standards (at each concentration) were plotted against the corresponding standard concentrations (in μg/mL) using weighted linear regression to generate a standard curve.

### Determination of terpene profile of the extracts by gas chromatography coupled with flame ionization or mass detector

The extracts were dissolved in methanol at a concentration of 2 mg/mL, transferred to the vial, and submitted to the GC-FID or GC-MS analysis.

To identify the terpenic profile, the cannabis extracts were analyzed by GC 7200 coupled with 7890 B qTOF mass detector (Agilent). Separation of individual volatile components was performed on a HP-5MS column (30 ms, 0.25 mm i.d., 0.25 μm; Agilent). Helium was used as a carrier gas; the flow was set at 1 mL.min^–1^. The oven temperature program began at 60°C with a 3.5 min hold; the temperature was increased at a rate of 3.5°C/min to 155°C and then again at a 30°C min^–1^ rate to 300°C and held for 10 min (total run time: 45 min). The temperature of the quadrupole was maintained at 230°C and of the ion source at 230°C. The compounds were measured in a scan mode in a range of 55–700 Da. The retention indices of each analyte were calculated from the retention times of n-alkanes by linear interpolation as previously described by Kováts. Identification of the analyzed volatiles was carried out by comparing the spectra with the spectra of available standards and/or by comparing their spectra and retention indices with the NIST Mass Spectral Search Program, version 2.2. The relative quantification of terpenes was performed by 7890A GC coupled with FID detector (Agilent) under the same chromatographic conditions. The detector conditions were set up as follows: *t* = 300°C; flow of gases - air: 400 mL/min; H_2_: 30 mL/min; and N_2_ make up flow: 5 mL/min. All samples were analyzed in triplicate.

### Determination of antimicrobial and antifungal activity of extracts

#### Tested microorganisms

The antibacterial activity of the extracts was tested against seven bacterial strains, namely, *Staphylococcus aureus* ATCC 25923 and 29213, *S. epidermidis* CCM 50, *S. saprophyticus* CCM 2727, *S. lugdunensis* CCM 4069, *S. epidermidis* CCM 4418, *Streptococcus pyogenes* CCM 4425. Their antifungal activity was determined against 12 dermatophytes, namely 3 strains of *Nannizzia fulva* (CCF 6025; 5338; 5782) two strains of *Trichophyton rubrum (CCF 4934*; *4879*, *Arthroderma insingulare* (CCF 5417; 5943), *Trichophyton tonsurans* CCF 4930, *Nannizzia gypsea* CCF 5215, *Epidermophyton floccosum* CCM 8339, *Microsporum canis* CCM 8353, and *Trichophyton interdigitale* CCM 8377. The strains were obtained from the American Type Culture Collection (ATCC), Czech Collection of Microorganisms (CCM) or Culture Collection of Fungi, Department of Botany, Charles University, Prague (CCF).

#### Broth microdilution method

To determine antimicrobial and antifungal activity expressed as minimal inhibitory concentration (MIC), slightly modified broth microdilution methods CLSI (CLSI, 2008, 2009) were used for bacteria and fungi, respectively. Both experiments were carried out in 3 technical and 3 independent replicates.

#### Determination of minimal inhibitory concentration for bacteria

The crude extracts were dissolved in DMSO to prepare the stock solution (*c* = 51.2 mg/mL). Subsequently, the two-fold serial dilution of the extracts was prepared at concentrations ranging from 4 to 512 μg/mL was prepared to 96 microtiter plates containing MHB as growth medium. In case of *S. pyogenes*, the MHB was supplemented with 7% horse defibrinated blood. The microplates were inoculated with bacteria at a final density of 0.5 McF (i.e., 1.5 × 10^8^ CFU/mL). The inocula were prepared from 1 day old bacterial cultures cultivated in MHB at 37°C. The degree of inhibition of bacterial growth was evaluated after 24 h cultivation at 37°C using the BioTek Synergy H1 microplate reader (Agilent) at 512 nm. The lowest concentration that inhibited bacterial growth by 80% compared to the growth control was considered the MIC. Chloramphenicol and ampicilin were used as positive controls.

#### Determination of minimal inhibitory concentration for dermatophytes

The stock solution of the extracts (*c* = 51.2 mg/mL) was by two-fold serial dilution dosed to 96 microtiter plates, resulting in concentrations ranging from 8 to 1,024 μg/mL. RPMI 1640 medium with pH 7 was used as growth medium. The microplates were inoculated with dermatophytes at density 3–7 × 10^5^ CFU/mL. Dermatophyte inocula were prepared from 14-day old fungal cultures grown on SDA at 27°C. The degree of fungal growth inhibition was evaluated after 5 days cultivation at 27°C by microplate reader BioTek Synergy H1 at 495 nm. The lowest concentration that inhibited the growth of the dermatophyte by 80% compared to the control was considered MIC. Terbinafine and clotrimazole were used as positive controls.

### Statistical evaluation of the data

The data were processed in Excel and STATISTICA 12 software (StatSoft, Tulsa, United States). As the data did not show normal distribution, the significant differences were evaluated by non-parametric Kruskal-Wallis test.

## Results and discussion

Most of the extracts prepared from two cannabis strains by three different solvents were predominantly (>60%) composed of various cannabinoids, especially THCA. All of them demonstrated activity against 18 of the 19 microorganisms tested. In general, the bacteria were more susceptible to the extracts in comparison to dermatophytes. The MICs of the extracts ranged from 4 to 256 μg/mL. The ethanolic ones were, in general, less potent than the DME and BUT ones. This fact can be attributed to lower content of the active compounds in EtOH extracts.

### Yield and chemical profile of cannabis extracts

The extraction yield by both gases was on average 4% higher compared to traditional maceration in ethanol (Table 1). The color of BUT and DME extracts was golden, while EtOH extracts were dark green. This indicates a higher content of chlorophyll content in EtOH extracts. High variability in cannabinoid content was observed if different solvents were used for the extraction of a single strain (Table 1). The most significant differences in the cannabinoid composition were determined in extracts prepared with ethanol. They contained less than half of the cannabinoids compared to the BUT and DME extracts (that is, CBGA ranged from 5.7 to 9.5 mg/g and 19 to 21 mg/g in EtOH and BUT/DME extracts, respectively (Table 1). The THC content in all extracts was similar. In general, the predominant cannabinoid in all extracts was THCA (190–617 mg/g), while the least present was THCV which was found only in Chocolope EtOH extract. The lower content of cannabinoids in EtOH extracts can probably be attributed to the higher polarity of the solvent and thus lower extraction ability of these compounds into the resulting extract (Politi et al., 2008; Romano and Hazekamp, 2013).

**TABLE 1:** Extract yield (%) and average cannabinoid content (mg/g) in extracts prepared by different solvents.

Cannabis strain	Forbidden fruit			Chocolope		
		
Solvent	EtOH	BUT	DME	EtOH	BUT	DME
CBC	1.35 ± 0.12^a^	2.67 ± 0.09^b^	5.84 ± 0.20^c^	1.45 ± 0.34^a^	2.69 ± 0.11^b^	2.42 ± 0.67^ab^
CBDA	3.22 ± 0.11^a^	11.82 ± 0.06^b^	12.78 ± 0.11^c^	4.01 ± 0.21^a^	13.08 ± 0.29^b^	13.74 ± 0.39^b^
CBG	0.54 ± 0.04^a^	2.37 ± 0.12^b^	3.01 ± 0.06^c^	1.05 ± 0.04^a^	2.66 ± 0.16^b^	2.32 ± 0.12^c^
CBGA	5.79 ± 0.17^a^	19.21 ± 0.78^b^	21.40 ± 0.81^c^	9.51 ± 0.22^a^	21.42 ± 0.75^b^	19.08 ± 0.56^c^
THC	35.24 ± 0.83^a^	43.29 ± 1.21^b^	41.50 ± 1.60^b^	51.23 ± 1.59^a^	46.28 ± 0.40^b^	58.50 ± 2.41^c^
THCA	199.80 ± 6.11^a^	576.65 ± 22.05^b^	581.58 ± 15.59^b^	261.75 ± 8.20^a^	613.53 ± 17.88^b^	617.68 ± 14.24^b^
THCV	ND	ND	ND	0.08 ± 0.02	ND	ND
TOTAL CANNABINOIDS	245.94 ± 7.3	656.01 ± 18.64	666.11 ± 16.84	329.10 ± 10.33	699.66 ± 18.64	713.73 ± 17.23
Extract yield (%)	10.5	13.84	14	11.18	13.7	14.1

Results are presented as mean ± standard deviation. The letters indicate statistically significant difference (p ≤ 0.05) in the cannabinod content within one cannabis strain extracted by different solvents. CBG, cannabigerol; CBGA, cannabigerolic acid; CBD, cannabidiol; CBDA, cannabidiolic acid; CBC, cannabichromene; THCV, tetrahydrocannabivarin; THC, Δ^9^-tetrahydrocannabinol; THCA-A, tetrahydrocannabinolic acid; EtOH, ethanol; BUT, butane; DME, dimethylether; ND, not detected.

The higher variability in the terpene profile was observed between strains and between extracts of one strain prepared with different solvents (Table 2). The main terpenes (>7% each) in Forbidden Fruit extracts were ß-caryophyllene, ß-myrcene, selina-4(15),7(11)-diene, selina-3,7(11)-diene followed by humulene and ß-farnesene. The Chocolope strain extracts contained mostly ß-caryophyllene, ß-myrcene, (+)-limonene, linalool, and selina-3,7(11)-diene. The results indicate that both strains belong to caryophyllene-dominated cultivars (Lewis et al., 2018), which are also characterized by relatively high levels of humulene (Fischedick, 2017) and low levels of the often predominant α-pinene (Lewis et al., 2018).

**TABLE 2:** Relative ratio (%) of terpenes in cannabis extracts prepared by different solvents.

	Forbiden Fruit			Chocolope			KI calculated	KI literature
		
Terpenes/solvent	EtOH	BUT	DME	EtOH	BUT	DME		
Thujene	ND	1.97 ± 0.05	6.14 ± 0.03	ND	0.99 ± 0.003	1.12 ± 0.01	931	929-930
α-pinene	ND	1.55 ± 0.03	3.4 ± 0.04	ND	1.81 ± 0.003	1.2 ± 0.02	974	942
ß-myrcene	ND	12.09 ± 0.28	9.54 ± 0.16	ND	8.34 ± 0.11	11.61 ± 0.08	990	**992**
(+)-Limonene	ND	2.94 ± 0.07	3.09 ± 0.04	0.59 ± 0.01	7.93 ± 0.03	9.12 ± 0.07	1027	1031
Linalool	1.46 ± 0.08^b^	1.24 ± 0.19^ab^	1.01 ± 0.005^a^	7.35 ± 0.02^b^	6.48 ± 0.05^ab^	6.03 ± 0.03^a^	1097	1100
Fenchol	0.62 ± 0.01	0.57 ± 0.02	0.61 ± 0.03	1.93 ± 0.01^b^	1.43 ± 0.01^ab^	1.21 ± 0.01^a^	1111	1119
Trans-2-pinanol	0.45 ± 0.02	0.4 ± 0.038	0.44 ± 0.02	1.45 ± 0.01^b^	1.02 ± 0.001^ab^	0.89 ± 0.01^a^	1119	1121
α-terpineol	0.86 ± 0.16	0.54 ± 0.04	0.57 ± 0.1	2.07 ± 0.01^b^	1.5 ± 0.01^ab^	1.19 ± 0.01^a^	1189	1185
ß-caryophyllene	**15.14 ± 0.08^ab^**	**14.62 ± 0.14^a^**	**18.97 ± 0.21^b^**	**14.87 ± 0.05^b^**	**11.49 ± 0.03^ab^**	**11.23 ± 0.09^a^**	**1418**	**1417**
α-bergamotene	3.75 ± 0.01^b^	2.78 ± 0.05^a^	2.9 ± 0.08^ab^	4.01 ± 0.11^b^	2.75 ± 0.01^ab^	2.7 ± 0.02^a^	1437	1434
humulene	**5.17 ± 0.02^ab^**	**4.53 ± 0.04^a^**	**6.31 ± 0.07^b^**	**5.02 ± 0.06^b^**	**3.72 ± 0.03^ab^**	**3.62 ± 0.0003^a^**	**1451**	**1437**
ß-farnesene	**6.37 ± 0.04^b^**	**4.4 ± 0.04^a^**	**4.44 ± 0.04^ab^**	**7.49 ± 0.1**	**4.96 ± 0.0004**	**5.01 ± 0.05**	**1456**	**1439**
γ-muurolene	0.62 ± 0.04^b^	0.47 ± 0.00^ab^3	0.4 ± 0.03^a^	0.37 ± 0.00	0.29 ± 0.01	0.3 ± 0.02	1475	1477
Aromadendrene	1.88 ± 0.02	1.35 ± 0.01	1.33 ± 0.04	1.43 ± 0.01	1.12 ± 0.14	1.12 ± 0.26	1485	1485
γ-selinene	2.01 ± 0.006	1.68 ± 0.02	1.64 ± 0.02	1.44 ± 0.08^b^	1.04 ± 0.005^a^	1.12 ± 0.01^ab^	1493	1484
δ-cadinene	1.14 ± 0.006	1.09 ± 0.06	0.79 ± 0.01	0.45 ± 0.003^b^	0.44 ± 0.04^ab^	0.48 ± 0.03^a^	1518	1520
α-cadinene	1.84 ± 0.02	2.39 ± 0.02	2.34 ± 0.03	1.11 ± 0.004^b^	0.76 ± 0.001^a^	1.06 ± 0.01^ab^	1539	1536
Selina-4(15), 7(11)-diene	**7.48 ± 0.07^ab^**	**7.95 ± 0.07^a^**	**5.55 ± 0.07^b^**	**2.5 ± 0.02^b^**	**3.64 ± 0.01^ab^**	**3.83 ± 0.03^a^**	**1540**	**1542**
Selina-3,7(11)-diene	**10.52 ± 0.12^ab^**	**12.23 ± 0.11^a^**	**9.01 ± 0.12^b^**	**3.06 ± 0.09**	**5.4 ± 0.01**	**5.34 ± 0.04**	**1543**	**1539**
Germacrene B	2.28 ± 0.01^b^	3.16 ± 0.01^a^	2.75 ± 0.32^ab^	1.55 ± 0.13^b^	3.43 ± 0.01^ab^	3.49 ± 0.05^a^	1559	1558
Guaiol	3.31 ± 0.03^b^	2.02 ± 0.02^ab^	0.61 ± 0.01^a^	5.31 ± 0.06^b^	3.67 ± 0.001^ab^	3.21 ± 0.03^a^	1596	1597
Humulene epoxide	0.75 ± 0.02^b^	0.23 ± 0.002^a^	0.35 ± 0.0003^ab^	2.12 ± 0.06^b^	1.06 ± 0.004^ab^	0.88 ± 0.01^a^	1610	1593
γ-eudesmol	3.93 ± 0.03^ab^	2.51 ± 0.03^a^	1.33 ± 0.02^b^	5.73 ± 0.03^b^	3.67 ± 0.005^a^	3.32 ± 0.03^ab^	1625	1635
ß-eudesmol	5.12 ± 0.03^b^	1.42 ± 0.27^ab^	0.74 ± 0.03^a^	3.8 ± 0.01^b^	1.84 ± 0.004^ab^	1.64 ± 0.02^a^	1659	1652
α-eudesmol	0.88 ± 0.03^b^	2.04 ± 0.12^ab^	0.55 ± 0.01^a^	1.21 ± 0.06^b^	3.29 ± 0.002^ab^	2.87 ± 0.02^a^	1674	1652
α-bisabolol	4.66 ± 0.03^b^	2.73 ± 0.03^a^	2.93 ± 0.03^ab^	1.12 ± 0.46^b^	0.91 ± 0.19^ab^	0.1 ± 0.004^a^	1688	1688
Juniper camphor	1.12 ± 0.001^b^	0.97 ± 0.01^ab^	0.9 ± 0.01^a^	0.47 ± 0.002^b^	0.34 ± 0.002^ab^	0.29 ± 0.01^a^	1699	1690

Results are expressed as mean ± standard deviation. DME, dimethylether; BUT, butane; EtOH, ethanol; ND, not detected; KI, Kovats index. The letters indicate statistically significant difference (p ≤ 0.05) in the cannabinod content within one cannabis strain extracted by different solvents. Major compounds are in bold letters.

The solvents had a statistically significant effect on the presence and relative ratio of individual terpenes in the extracts (Table 2). Both EtOH extracts compared to BUT and DME ones lacked most of the identified monoterpenes (i.e., thujene, α-pinene, ß-myrcene, and (+)-limonene). These terpenes were probably lost during the evaporation of the solvent under vacuum and increased temperature (Romano and Hazekamp, 2013). In conclusion, the gas extraction was more effective, BUT and DME extracts contained significantly higher amounts of active compounds. On top of that, the extraction was less time demanding (did not require the solvent evaporation step). Moreover, DME is a non-toxic gas commercially used for collagen extraction (EFSA, 2015). Therefore, it could be considered as a suitable alternative to ethanolic extraction.

### Antifungal and antibacterial activity of extracts

All extracts showed very good to moderate activity against 18 of the 19 microorganisms tested. The MIC of the extracts ranged from 4 to 128 μg/mL and 32 to 256 μg/mL (Table 3) for bacteria and dermatophytes, respectively.

**TABLE 3:** Minimal inhibitory concentrations (MIC) (μg/mL) of cannabis extracts against selected bacteria and dermatophytes.

	Microorganism	Strain	MIC (μg.mL^–1^)							
			
			Forbidden fruit			Chocolope			Antibiotics	
					
			EtOH	BUT	DME	EtOH	BUT	DME	CLT	TB
dermatophytes	*Arthroderma insingulare*	CCF 5417	256	128	128	256	64	64	0.5	2
		CCF 5943	>1024	>1024	>1024	>1024	>1024	>1024	1	0.5
	*Epidermophyton floccosum*	CCM 8339	64	64	64	128	64	64	16	>16
	*Microsporum canis*	CCM 8353	128	128	128	256	128	128	0.25	0.125
	*Nannizzia fulva*	CCF 6025	64	32	32	64	32	32	0.0313	2
		CCF 5338	128	64	64	128	64	64	0.0625	2
		CCF 5782	128	64	64	128	64	64	0.125	4
	*Nannizzia gypsea*	CCF 5215	128	64	64	128	64	64	0.0625	0.125
	*Trichophyton interdigitale*	CCM 8337	64	64	64	128	64	64	0.0625	0.25
	*Trichophyton rubrum*	CCF 4934	256	128	128	256	128	128	1	0.5
		CCF 4879	128	32	32	128	16	16	1	0.125
	*Trichophyton tonsurans*	CCF 4930	128	64	64	128	64	64	1	0.125
bacteria			EtOH	BUT	DME	EtOH	BUT	DME	AMP	CLP
	*Staphylococcus aureus*	ATCC 29213	8	4	4	8	4	4	1	8
		ATCC 25923	8	4	4	8	4	4	0.0625	8
	*Staphylococcus epidermidis*	CCM 50	16	8	16	8	8	16	0.0625	6.25
		CCM 4418	8	8	8	8	8	8	2	3.125
	*Staphylococcus lugdunensis*	CCM 4069	16	16	16	16	16	16	0.25	1.56
	*Staphylococcus saprophyticus*	CCM 2727	8	4	4	8	16	16	0.5	3.125
	*Streptococcus pyogenes*	CCM 4425	128	64	64	128	64	64	16	3.125

DME, dimethyl ether; BUT, butane; EtOH, ethanol; AMP, ampicillin; CLP, chloramphenicol; CLT, clotrimazole; TB, terbinafine.

The most susceptible bacteria were *S. aureus* strains (MIC = 4–8 μg/mL). However, almost 10 times higher MIC values (MIC = 64–128 μg/mL) were determined for *S. pyogenes*. Such a significant decrease in the activity of cannabis extracts could be attributed to the presence of blood in the cultivation medium. As previously reported, in this type of media, cannabinoids become less effective due to their strong affinity to blood proteins (Garrett and Hunt, 1974; van Klingeren and Ten Ham, 1976). All extracts were also slightly less effective against *S. aureus* strains compared to isolated THC, CBD, CBG, and CBC (MIC = 0.5–2 μg/mL) (Appendino et al., 2008; Blaskovich et al., 2021). Generally, all the extracts except the EtOH ones were from more than 60% composed of cannabinoids (Table 1), therefore, they can be considered antimicrobial principles of cannabis. However, the role of the remaining biologically active compounds, especially terpenes, should not be omitted. The antimicrobial activity of the terpenes identified in extracts (Table 2) has been confirmed by many studies (Schofs et al., 2021). For example, ß-caryophyllene and α-pinene are very effective against *S. aureus*, *S. epidermidis*, *S. saprophyticus* and *S. lugdunensis* (Dahham et al., 2015; Ranarivelo et al., 2020). While myrcene has proven effects against *S. epidermidis* (Inoue et al., 2004) and limonene also has strong bactericidal effects against a diverse range of bacteria (Han et al., 2020, 2021).

The most sensitive dermatophytes were *T. rubrum* CCF 4879 (the most common species causing dermatophytosis in developed countries) and *M. canis* CCF 6025, while *A. insingulare* was resistant even to the highest concentrations of the extracts tested. The mode values of the MICs demonstrated that the EtOH extracts were slightly less effective (MIC = 128 μg/mL) compared to the DME and BUT extracts (MIC = 64 μg/mL). Even though some of the cannabis skin preparations are among other disorders also indicated for the treatment of bacterial and fungal skin diseases (Hashim et al., 2017; Anastassov et al., 2019), the scientific evidence about their activity against dermatophytes is very limited. So far, only Turner and Elsohly (1981) reported significant antifungal effects of CBC and its’ analogs against *Trichophyton mentagrophytes* (MIC = 6.25 – 50 μg/mL^–1^). Also hemp water extracts proved to have moderate activity against *T. rubrum* and *interdigitale* (MIC = 500 μg/mL) but not against *N. gypsea* (Orlando et al., 2020). There is no doubt that cannabinoids have a beneficial effect on various skin problems such as skin inflammation, fibrosis, itch, pain, and improved wound healing (Hashim et al., 2017; Cintosun et al., 2020). However, their contribution to the antifungal activity remains questionable and a possible mechanism of action is yet to be elucidated. On the other hand, all extracts contained terpenes with established activity against dermatophytes. For example, essential oils from cannabis with high concentrations of ß-caryophyllene or ß-caryophyllene alone were very effective against various dermatophytes (Iordache et al., 2016; Orlando et al., 2021). Tavares et al. (2010) reported a strong MIC (1–5 μg/mL) of myrcene against a variety of microorganisms, including dermatophytes. Moreover, limonene, α-pinene, γ-eudesmol, germacrene (separated or as a apart of essential oil) also proved significant to moderate antidermatophytic activity (MIC = 0.8–250 μg/mL) which was sometimes even better than some used antifungals, e.g., griseofulvin (Sanguinetti et al., 2007; Singh et al., 2010; Pinto et al., 2013; Alves et al., 2018; Danielli et al., 2018). It is very probable that both groups of biologically active substances, i.e., cannabinoids and terpenes, have an important role in the antifungal activity of cannabis extract.

The MIC values of cannabis extracts presented in this study are higher in comparison to the currently used antifungals. Furthermore, as mentioned above, some of the isolated compounds can be more active than the extracts. Therefore, the presented results may raise the question about the real therapeutic potential of cannabis extracts in the treatment of various skin infections. Current antimicrobial and antifungal agents were design solely to kill the target microorganism (Gnat et al., 2020). On the other hand, the effect of cannabis extract can be more complex. Not only it can inhibit or kill the microorganism growth, but both cannabinoids and terpenes due to their anti-inflammatory, antipruritics and antinociceptive properties can have positive effect on other symptoms accompanying the skin infections. Cannabinoids can also interact with the skin endocannabinoid system that plays an important role in its homeostasis (Martins et al., 2022). Terpenes are understood as skin penetration enhancers (Aqil et al., 2007), thus can facilitate the penetration of highly lipophilic cannabinoids to deeper skin layers or by other ways enhance their activity. This so called “entourage effect” was quite recently confirmed for some of the terpenes present in cannabis (LaVigne et al., 2021). From the empirical evidence provided by many users of cannabis topicals, it is known that the preparations are usually well accepted and reported to be effective (Martins et al., 2022). Although topicals are often homemade or from unofficial sources and contain unknown concentrations of various cannabis chemotypes (Mahmood et al., 2022). Therefore, their low toxicity during long-term use can be expected. These facts confirm that the therapeutic potential of cannabis in the treatment of skin infections should not be overlooked. Especially for the treatment of dermatomycosis that is usually long and can be associated with adverse effects. The side effects are unacceptable for elderly, pregnant women and children (Gnat et al., 2020).

To confirm the safety and efficacy of cannabis extracts that will lead to their use in medical practice, a lot of work has to be done. Among others identification of most active cannabis chemotypes, standardization of the extracts, determination of effective dose, pharmacokinetics, toxicological risk assessment and clinical studies are needed.

## Conclusion

All tested extracts demonstrated significant activity against 18 of 19 tested bacterial and fungal skin pathogens. To the best of our knowledge, this is the first report on anti-dermatophyte activity of high potency cannabis strains extracted by different solvents. The extracts prepared traditionally by maceration in ethanol had lower amounts of active compounds, which led to their lower activity against the tested microorganisms. While the extraction by gas (DME, BUT) was more effective and less laborious. Since DME is successfully used in food industry, its pharmaceutical and commercial potential for plant extraction should be further considered and investigated.

It is already known that cannabinoids have a relatively wide spectrum of biological activities for which they are useful in the treatment of skin diseases. However, not much attention has been paid to their effectiveness against bacteria and fungi, which are often accompanied by skin problems. Our research brought new evidence that cannabis extracts may be of value to treat dermatophytosis and other skin diseases caused by various microorganisms and showed that cannabis could serve as an alternative or supportive treatment to commonly used antibiotics.

## Data availability statement

The raw data supporting the conclusions of this article will be made available by the authors, without undue reservation.

## Author contributions

TS and ZK: writing – original draft and investigation. JT: investigation, validation, and writing – review and editing. AJ: resources. PK: writing – review and editing. VH: resources and writing – review and editing. AF: conceptualization, data curation, methodology, writing – original draft, review and editing, and supervision. All authors contributed to the article and approved the submitted version.
